# Child and Adolescent Psychiatry and Mental Health reviewer acknowledgement 2012

**DOI:** 10.1186/1753-2000-7-2

**Published:** 2013-02-22

**Authors:** Jörg M Fegert

**Affiliations:** 1 Department of Child and Adolescent Psychiatry and Psychotherapy, University of Ulm, Steinhoevelstr. 5, Ulm, 89075, Germany

## Abstract

**Contributing reviewers:**

The editors of *Child and Adolescent Psychiatry and Mental Health* would like to thank all of our reviewers who have contributed to the journal in volume 6 (2012).

## 

Howard Abikoff

United States of America

Gwen Adshead

United Kingdom

Marc Allroggen

Germany

Kim Anderson

United States of America

Muhammad Ayub

United Kingdom

Melanie Barwick

Canada

Vincenza Bianchi

Italy

Nicoletta Bisacchi

Italy

Jeffrey Bishop

United States of America

Margarete Bolten

Switzerland

Wolfgang Briegel

Germany

Thomas E Brown

United States of America

Belinda Bruwer

South Africa

Regina Bussing

United States of America

Marilyn Campbell

Australia

Luna Centifanti

United Kingdom

Patricia Chamberlain

United States of America

Alexander Chapman

Canada

Ying-Sheue Chen

Taiwan

Megan Chesin

United States of America

Ann Childress

United States of America

Molly Choate Summer

United States of America

Monali Chowdhury

United States of America

Penny Corkum

Canada

Samuele Cortese

United States of America

Michael Davidovitch

Israel

Domenico De Berardis

Italy

Mieke Decuyper

Belgium

Carmen Desirée Dirksen

Netherlands

Kathy Dowell

United States of America

Kimberly DuMont

United States of America

Michael Dunn

United Kingdom

Tony Durkee

Sweden

Mayada Elsabbagh

Canada

Jerome Endrass

Switzerland

Tatiana Falcone

United States of America

Dineke Feenstra

Netherlands

Minne Fekkes

Netherlands

David Fergusson

New Zealand

Reiner Frank

Germany

Jennifer Freeman

United States of America

Mark Friedman

United States of America

Barbara Friesen

United States of America

Sari Fröjd

Finland

Daniel Fung

Singapore

Abbe Garcia

United States of America

Morton Gernsbacher

United States of America

Catherine Glenn

United States of America

Marianne Goodman

United States of America

John Gunderson

United States of America

Penelope Hasking

Australia

Anders Helander

Sweden

Marisa Hilliard

United States of America

Megan Hobbs

Australia

Sumi Hoshiko

United States of America

Jennifer Hughes

United States of America

Stephanie Hullmann

United States of America

Tonje Lossius Husum

Norway

Joost Hutsebaut

Netherlands

Nicholas Ialongo

United States of America

Susan Jack

Canada

Astrid Janssens

United Kingdom

Lisa M Jones

United States of America

Michael Kaess

Germany

Riittakerttu Kaltiala-Heino

Finland

Nestor Damian Kapusta

Austria

Niranjan Karnik

United States of America

Yotaro Katsumata

Japan

Ferdinand Keller

Germany

Reinhold Kilian

Germany

Heinz Kindler

Germany

David J Klein

United States of America

Susanne Knappe

Germany

Denis Köhler

Germany

Maria Kovacs

United States of America

Kasia Kozlowska

Australia

E Stephanie Krauthamer Ewing

United States of America

Maya Krischer

Germany

Anne Kroeger

Germany

Caroline Kuo

United States of America

Sylvia Kwok

Hong Kong

Magali Lahaye

Belgium

Bo Larsson

Norway

Gerd Lehmkuhl

Germany

Ulrike Leiss

Austria

Adam Lewin

United States of America

Stephen P Lewis

Canada

Ramon Lindauer

Netherlands

Astri Lundervold

Norway

Jenny Macfie

United States of America

Ann Maloney

United States of America

Abigail Marsh

United States of America

Graham Martin

Australia

Andres Martin

United States of America

Erin McClure

United States of America

Bo Mohl

Denmark

Suneeta Monga

Canada

Jennifer Muehlenkamp

United States of America

Abraham Mukolo

United States of America

Wendy Nilsen

United States of America

Latha Nrugham

Norway

Beate Oerbeck

Norway

Peter O'Gorman

Ireland

Susanne Ohmann

Austria

Guiomar Oliveira

Portugal

Ukamaka Oruche

United States of America

Sonja Perren

Switzerland

John Piacentini

United States of America

Bettina Piko

Hungary

Dominic Plant

United Kingdom

Paul L Plener

Germany

Christian Postert

Germany

Roslyn Poulos

Australia

Colin Pritchard

United Kingdom

Steven Pryjmachuk

United Kingdom

Lamprini Psychogiou

United Kingdom

Naren Rao

Canada

Erin Reuther

United States of America

Wolfgang Retz

Germany

Carla Sabariego

Germany

Daniel Safer

United States of America

Mariacarolina Salerno

Italy

Benno Schimmelmann

Germany

Robert Schlack

Germany

Marc Schmid

Switzerland

Ulrike Margarete Elisabeth Schulze

Germany

Nadine Schwartz

United States of America

Emma Sciberras

Australia

Stefano Seri

United Kingdom

Carla Sharp

United States of America

Joel Sherrill

United States of America

Margaret Sibley

United States of America

Susan Silva

United States of America

Ellin Simon

Belgium

Teesta Soman

Canada

Lin Sørensen

Norway

Cesar Soutullo

Spain

Hans-Christoph Steinhausen

Denmark

Lisanne Stone

Netherlands

Eric Storch

United States of America

Jeffrey Stuewig

United States of America

Kevin Sullivan

United States of America

Calvin Sumner

United States of America

Anne Mari Sund

Norway

Sharon Sung

Singapore

Lene Symes

United States of America

Toshinobu Takeda

Japan

Jessica Tearne

Australia

Elaine Thompson

United States of America

Ute Thyen

Germany

Audrey Tluczek

United States of America

Lieke van Domburgh

Netherlands

Martijn van Noorden

Netherlands

Violaine Veen

Netherlands

Robert Vermeiren

Netherlands

Alexander von Gontard

Germany

Rebecca Vujnovic

United States of America

Jan Wallander

United States of America

Michelle Wedig

United States of America

Oliver White

United Kingdom

Silke Wiegand-Grefe

Germany

Patricia Williams

United States of America

Denise Wilson

New Zealand

Tuppett Yates

United States of America

Cheng-Fang Yen

Taiwan

